# Effectiveness of aloe fomentation for nipple-related complications during the early puerperium period: a randomized, controlled, interventional study

**DOI:** 10.1186/s13104-022-05980-x

**Published:** 2022-03-07

**Authors:** Yumiko Tateoka

**Affiliations:** Department of Nursing, Faculty of Nursing, Shiga University of Medical Science, Seta Tsukinowa-Cho, Otsu, Shiga 520-2192 Japan

**Keywords:** Aloe arborescens fomentation, Early puerperium period, Breastfeeding, Eschar, Nipple care

## Abstract

**Objective:**

Nipple-related complications are major factors that prevent breastfeeding for many new mothers. Hence, we tested the effects of aloe arborescens fomentation applied to the nipples as a treatment for nipple-related complications.

**Results:**

This study included 60 women who breastfed for the first time on day 1 after delivery. Every 24 h, all women breastfed six times and bottle-fed two times (at night). Women were classified into an intervention group (aloe arborescens fomentation) and a control group (no treatment). Aloe fomentation was applied after breastfeeding six times per day. We observed the nipples three times per day for 5 days after delivery. The most common nipple-related complication in this study was redness. A significant decrease was observed for women in the intervention group.

*Trial Registration* Retrospectively Registered to registry: UMIN; Registration no.: UMIN000044514; Registered on: 11th June 2021.

## Introduction

Breastfeeding during the first 6 months–1 year of life is not only beneficial for establishing a bond between the mother and child but also increases the child’s resistance against infectious diseases [1–3]. In Japan, the exclusive breastfeeding rate during the first month after birth was only 42.2% in 2005 [4]. Nipple-related complications, like pain associated with cracking, soreness, bleeding, or blisters, are among the major reasons behind the reluctance of mothers to breastfeed [5–8]. The preventive/interventional methods to reduce nipple pain and injured nipples include direct application of breast milk, lanolin or vitamin A ointment, warm water fomentation, tea bag fomentation, hydrogel dressing using nipple protectors, heat therapy, the clean and dry method [8, 9]. However, no method showed clear benefits.

Aloe has long been considered to have several medicinal benefits and is cultivated worldwide [10]. In Japan, it is used in medicinal products and is available without a prescription. A recent clinical study has suggested the effectiveness of aloe vera gel in healing nipple soreness [11]. Aloe arborescens, like aloe vera, is known for its succulent green leaves and used to treat a wide variety of ailments because of its antitumor, anti-inflammatory, and wound-healing effects [12, 13].

In this study, with the intention of finding a new method of mammary care, it was hypothesized that applying aloe to the nipples might be an effective prevention/intervention for nipple-related complications of nursing women. Application of a wet fomentation was shown to protect the nipples from external irritation due to friction during the early puerperium period. As aloe leaf contains 95.5% water [14], we aimed to assess if the aloe components or the gel inside the leaf could be used as a potential fomentation medium. This study tested application of aloe fomentation as a method of caring for nipple-related complications.

## Main text

### Methods

#### Participants

Sixty-five women were invited to participate in the study. Four women were excluded because either the mother or the child had an abnormality during the 5-day period after birth. Another woman decided not to participate. Therefore, we included 60 women who had just given birth and were breastfeeding for the first time on day 1 post-birth in this randomized, controlled, interventional study between May 2008 and July 2008. The included women were aged 20 years or older, had a single vaginal term birth, and agreed to breastfeed six times and bottle-feed two times (at night) every 24 h. Women were included in the study following verbal and textual explanations before day 1 post-birth. Written consent was obtained from the women, and a questionnaire was provided. We excluded mothers who had medical diseases or nipple injuries and those who gave birth to premature infants. We split the women into two groups: a control group that did not receive any treatment and an intervention group that received aloe fomentation to apply to the nipple after breastfeeding. Differences due to factors influencing nipple-related complications other than the intervention methods were considered to ensure consistency across breastfeeding instructions for women. The same researchers provided lactation guidance beginning 24 h after childbirth. We provided instructions for breastfeeding based on a pamphlet about breastfeeding methods for approximately 20 min before the woman breastfed for the first time on day 1 post-birth.

#### Study procedure

Each woman selected for the intervention group underwent a preliminary patch test. None of the women had any allergic reactions, such as redness or itching of the skin. After completing breastfeeding for the first time, aloe fomentation was applied to the nipples of the women in the intervention group. The remaining aloe fomentation was then stored in the refrigerator. Methods for applying the aloe fomentation on the nipples and suitable storage were explained to the women. The nipples and areola were cleansed with clean cotton and purified water before the next breastfeeding session to completely remove the aloe and ensure that the aloe fomentation had no influence on the child.

At the research institute of the study, infants were breastfed six times each day at 3-h intervals (bottle-feeding was performed twice at night). The study design was such that the frequency of breastfeeding every day and the breastfeeding period were constant for all women.

We completed the study survey based on the women’s basic information and observations of the women’s nipples and breasts. The nipples and breasts were observed three times per day (before breastfeeding at 10:00, 13:00, and 16:00) from day 1 to day 5 after birth when the women were discharged. Nipple-related complications were graded as “redness of the nipple/areola,” “fissure of the tip, sidewall, or neck of the nipple,” “nipple epidermolysis,” “edema of the nipple/areola,” “bleeding from the nipple,” “nipple eschar,” “water blister at the nipple pore,” or “blood blister at the nipple pore.” This study adheres to CONSORT guidelines.

#### Preparation of the aloe fomentation

Aloe arborescens leaves were obtained from Aloe Center (Izu, Japan) and thoroughly washed and peeled. The transparent gel was removed, sterilized in boiling water, and then prepared for application [11].

### Statistical analysis

Data are expressed as mean ± standard deviation. Microsoft Office Excel 2007 (Redmond, WA, USA) and SPSS 11.5 J for Windows (Chicago, IL, USA) were used for statistical processing.

#### Ethics approval and consent to participate

This study was approved by the Ethics Review Subcommittee of the Department of Health Sciences, Nagoya University School of Medicine. (Approval number 7–167). Written consent was obtained from the participants.

### Results

Among the 60 study participants, aloe poultices made into 3 × 3 cm squares were applied to 30 women in the intervention group, and the remaining 30 women served as the control group. The women’s basic information and information at the time of childbirth are shown in Table 1. There were no significant differences among the women regarding the characteristics considered.

**Table 1:** Characteristics of the women

	Intervention group (n = 30)	Control group (n = 30)
	Mean ± SD	Mean ± SD
Age, years	28.4 ± 4.6	29.3 ± 5.5
Time required for childbirth, minutes	756.6 ± 464.6	714.8 ± 405.4
Intrapartum hemorrhage, g	190 ± 95.7	264 ± 169.9
Birth weight, g	2902.1 ± 304.7	2921.9 ± 271.4
		N = 60

#### Incidence rate of nipple-related complications

Observations were performed three times per day, and nipple-related complications were considered present if they were observed at least once that day. The incidence rates for the left nipple increased until day 4 and peaked at 63.3% in the control group, but they did not increase beyond 43.3% in the intervention group. There was no statistically significant difference between the control and intervention groups regarding the left nipple.

The incidence rate of right nipple-related complications, excluding day 1 post-birth, was higher in the control group compared to the intervention group. The difference became statistically significant on days 4 and 5 post-birth, when the rate started to decline and stabilized at 26.7% (p < 0.05) in the intervention group. This indicated that the aloe fomentation was effective for right nipple-related complications.

#### Incidence of different nipple-related complications

**Fig. 1:**
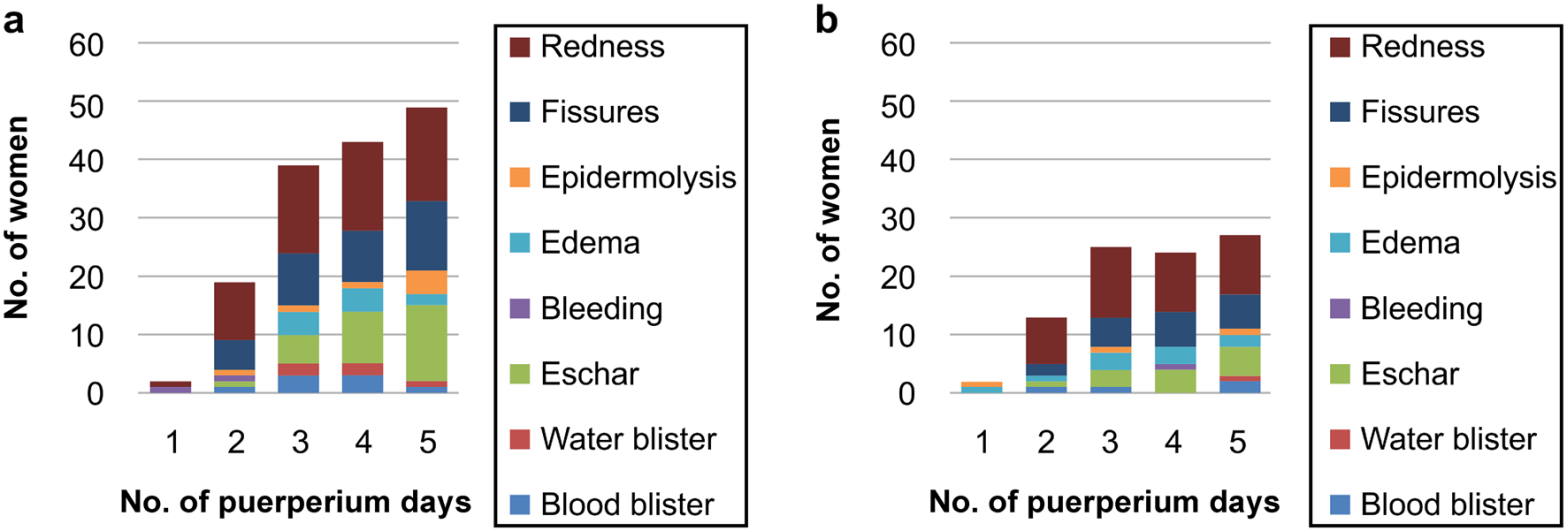
Incidence of nipple-related complications in **a** the control group and **b** the intervention group

Subsequently, we studied the incidence of each type of nipple-related complication experienced by the women (Fig. 1). Each complication was considered as one entry; it was possible for the women to have multiple entries of nipple-related complications. From day 2 post-birth, the number and type of nipple-related complications increased in both groups. Among the nipple-related complications for all women, the most common was redness (38 women), followed by fissures (26 women), eschar (21 women), epidermolysis (9 women), edema (9 women), water blisters (6 women), blood blisters (5 women), and bleeding (4 women).

**Fig. 2:**
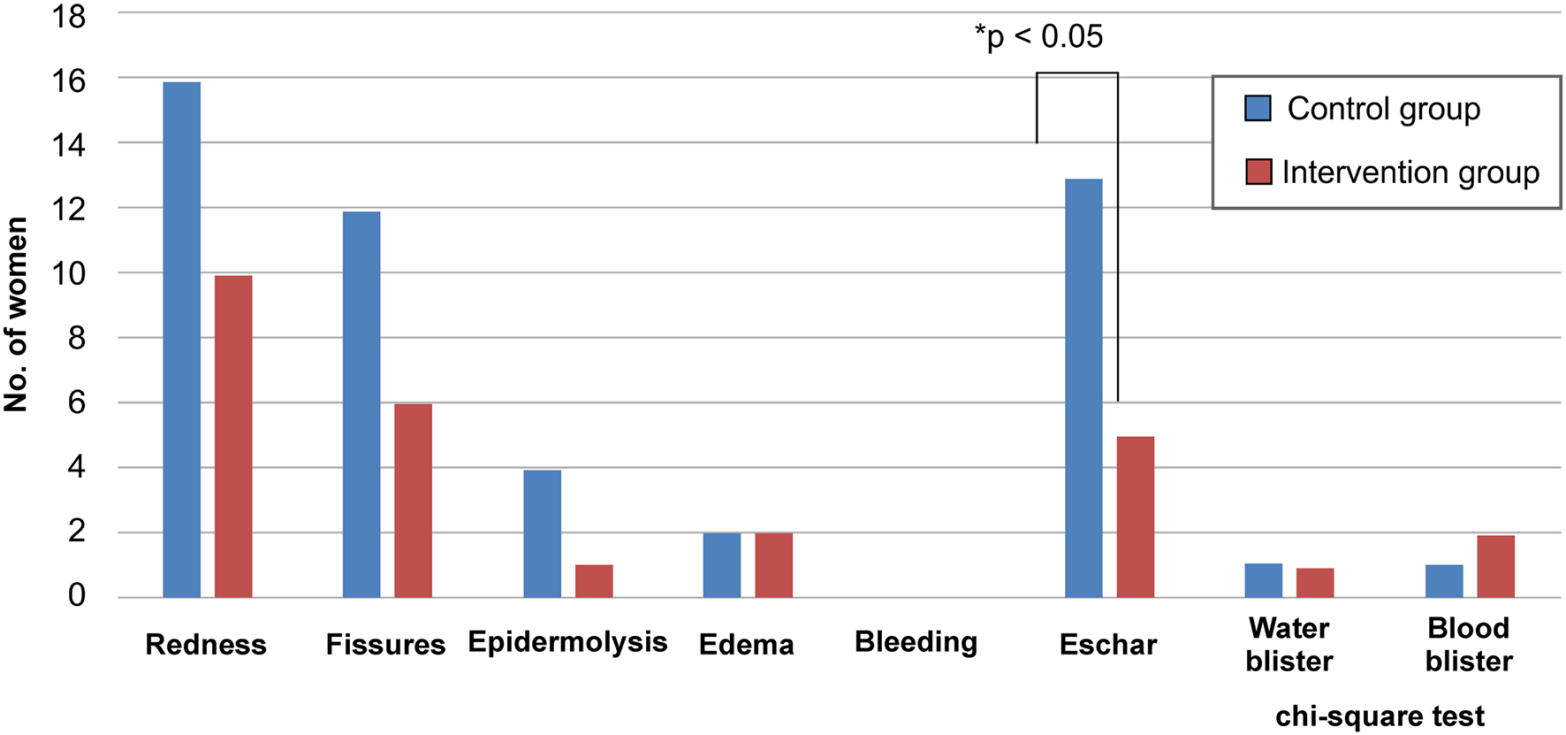
Incidence of nipple-related complications on day 5 post-birth

Statistically, there was no significant difference between the intervention and control group for redness, epidermolysis, edema, or bleeding. However, from day 2 to day 5 post-birth, the incidence of redness was higher in the control group. A comparison between the control and intervention group regarding the incidence of nipple-related complications on day 5 post-birth (the last day of the study) is shown in Fig. 2. There was a statistically significant difference for eschar on the last day of the study, which was day 5 post-birth (Fig. 2; p < 0.05). Therefore, among all nipple-related complications, aloe fomentation was the most effective for the incidence of eschar.

### Discussion

#### Nipple-related complications during the early puerperium period

We used the term “fissure of the nipple” for wounds or fissures on the nipple skin. However, the terminologies for various complications have been used inconsistently throughout reports, making comparisons difficult [15]. In our study, we accounted for different nipple-related complications and recorded multiple complications for each woman. Previous studies discussed the course and symptoms of nipple-related complications and how the breakdown of the bullae epidermis caused peeling of the skin, resulting in dermal fissures or microhemorrhage and eschar or occasional pus droplet formation [16, 17]. However, our study showed that women who presented with water blisters might not have had epidermolysis or fissures. It is apparent that fissures may not be followed by hemorrhage and may not progress into an eschar. The results of this study indicated that depending on the woman, there were multiple nipple-related complications that did not necessarily occur in any particular sequence.

#### Intervention methods for avoiding nipple-related complications

Researchers studied the comparative effectiveness of using a hot compress, fresh breast milk, and cleanliness for the prevention and relief of pain and fissures on the nipple in women who gave birth for the first time [18]. The incidence of nipple fissures was the highest on day 3 post-birth for women who used hot compresses and fresh breast milk. To prevent the problem in previous study design that did not include autonomous breastfeeding, we planned to verify the effect of aloe fomentation for nipple-related complications of women who autonomously breastfeed.

#### Effect of aloe fomentation on nipple-related complications

There are instances when symptoms of nipple-related complications worsen, resulting in an infection in the breast or nipple [19]. The results of this study showed that aloe fomentation applied to the nipples was effective for decreasing nipple-related complications. Aloenin and barbaloin, the main components of aloe, have inhibitory effects on histamine discharged from the mast cells [20]. Anthraquinone and anthranilic acids in the aloe vera extract inhibit endogenous prostaglandin biosynthesis, which is involved in the onset of pain [10]. Yamamoto et al. [21] reported that aloe arborescens components also have anti-inflammatory effects.

Among the nipple-related complications, there was a significant difference in the incidence of eschar on the nipple between the intervention and control group. A previous study indicated that Piyabayu could have been more effective in treating nipple-related complications, if it had been applied before the onset of complications [22]. As preventive approach for complications is important in obstetrics care, we started using an aloe fomentation at the outset, before any complications were witnessed.

One of the causes of nipple-related complications was pressure from wearing an undergarment like a bra or nursing pad [23], causing physical irritation. The application of aloe fomentation to the nipples after breastfeeding helped prevent direct contact between the nipple and nursing pad or undergarment to reduce physical irritation.

It is indicated that applying aloe to the skin results in contact dermatitis due to allergic reactions or irritation [24–29]. To lower the rate of contact dermatitis, instead of applying the juice of the aloe leaf, we used the gel inside the leaf for aloe fomentation in the study and confirmed this by performing a patch test prior to the start of the study. We concluded that an aloe arborescens fomentation might be effective for preventing aggravation of nipple-related complications especially the incidence of eschar.

## Limitations

•We did not investigate effective components of our aloe arborescens fomentation; these components are something that future studies could address.
•The anti-inflammatory effects of aloe fomentation for nipple-related complications could not be clarified because the study period lasted only from 1 to 5 days post-birth. Longer studies would be helpful in determining whether aloe fomentation could be effective in such cases.

## Data Availability

The datasets used and/or analyzed during the current study are available from the corresponding author on reasonable request.

## References

[1] Schmied V, Barclay L (1999). Connection and pleasure, disruption and distress: women’s experience of breastfeeding. J Hum Lact.

[2] Wrigley EA, Hutchinson SA (1990). Long-term breastfeeding: the secret bond. J Nurse Midwifery.

[3] Kramer MS, Kakuma R (2012). Optimal duration of exclusive breastfeeding. Cochrane Database Syst Rev.

[4] Inoue M, Binns CW, Otsuka K, Jimba M, Matsubara M (2012). Infant feeding practices and breastfeeding duration in Japan: a review. Int Breastfeed J.

[5] Beppu Y, Kumamoto K, Miyaoka M, Ogasahara M, Kouno M, Samei K (2006). A study on the inhibitory factors that prevent breast feeding. Nihon Josan Gakkai.

[6] Wakita H, Harada M, Kimoto M, Wada K, Shimoi K, Minamitake H (2006). The factors hindering breast feeding and the counter measures. Kagoshima J Mater Health.

[7] Dennis CL, Jackson K, Watson J (2014). Interventions for treating painful nipples among breastfeeding women. Cochrane Database Syst Rev.

[8] Morland-Schultz K, Hill PD (2005). Prevention of and therapies for nipple pain: a systematic review. J Obstet Gynecol Neonatal Nurs.

[9] Buchko BL, Pugh LC, Bishop BA, Cochran JF, Smith LR, Lerew DJ (1994). Comfort measures in breastfeeding, primiparous women. J Obstet Gynecol Neonatal Nurs.

[10] Yagi A (1993). Efficacy and effect of aloe. Annual report of Faculty of Pharmacy & Pharmaceutical Sciences.

[11] Saeidi R, Tafazoli M, Gholami M, Mazloom R (2015). New treatment for nipple soreness in breastfeeding mothers: a clinical trial study. Iran J Neonatol.

[12] Singab AN, El-Hefnawy HM, Esmat A, Gad HA, Nazeam JA (2015). A systemic review on aloe arborescens pharmacological profile: biological activities and pilot clinical trials. Phytother Res.

[13] Jia Y, Zhao G, Jia J (2008). Preliminary evaluation: the effects of *Aloe**ferox* Miller and *Aloe**arborescens* Miller on wound healing. J Ethnopharmacol.

[14] Yasuka T (1986). Cosmetic dermatology aloe update. Pract Dermatol.

[15] Yagi T, Tateoka Y, Yamashita M (2009). History of coping strategies for fissures of nipples in Japan. J Jpn Soc Breastfeed Res.

[16] Magara M (1993). Obstetrics: abnormal. Edition (revision draft 19). Obstetrics Science.

[17] Friedman E, Green Hill JP (1980). Obstetrics. (Suzuki M, Trans).

[18] Akkuzu G, Taşkin L (2000). Impacts of breast-care techniques on prevention of possible postpartum nipple problems. Prof Care Mother Child.

[19] Marchant DJ (2002). Inflammation of the breast. Obstet Gynecol Clin North Am.

[20] Nakagomi K, Yamamoto M, Tanaka H, Tomizuka N, Masui T, Nakazawa H (1987). Inhibition by aloenin and barbaloin of histamine release from rat peritoneal mast cells. Agric Biol Chem.

[21] Yamamoto M, Masui T, Sugiyama K, Yokota M, Nakagomi K, Nakazawa H (1991). Anti-inflammatory active constituents of aloe arborescens Miller. Agric Biol Chem.

[22] Hara K, Nakao Y, Yamamoto N, Oishi K (2007). The effect of Piabayu on nipple injury during puerperium. Health Sci J.

[23] Nezu H (1997). Breast management studies.

[24] Imagawa H, Tomonori O, Tsunaro O (2000). Contact dermatitis resulting from clematis-reported cases of skin problems in Japan due to home remedies. Pract Dermatol.

[25] Kubo Y, Nonaka K, Yoshida H (1987). Irritant contact dermatitis due to aloe. Skin Res.

[26] Nakamura T (1984). Phytodermatitis. Pract Dermatol.

[27] Sashida Y (1984). 1 case of phytodermatitis due to aloe. Pharmaceutics.

[28] Shoji A (1975). 1 case of contact dermatitis due to aloe arborescens. Jpn J Clin Dermatol.

[29] Yamada Y, Suzuki K, Jidoi J (1995). Contact dermatitis due to aloe. Pract Dermatol.

